# Histopathological Effects of Bt and TcdA Insecticidal Proteins on the Midgut Epithelium of Western Corn Rootworm Larvae (*Diabrotica virgifera virgifera*)

**DOI:** 10.3390/toxins9050156

**Published:** 2017-05-08

**Authors:** Andrew J. Bowling, Heather E. Pence, Huarong Li, Sek Yee Tan, Steven L. Evans, Kenneth E. Narva

**Affiliations:** Dow AgroSciences, Indianapolis, IN 46268, USA; hepence@dow.com (H.E.P.); hli2@dow.com (H.L.); STan5@dow.com (S.Y.T.); slevans@dow.com (S.L.E.); knarva@dow.com (K.E.N.)

**Keywords:** western corn rootworm, *Diabrotica virgifera virgifera*, histopathology, *Bacillus thuringiensis*, Cry34Ab1, Cry35Ab1

## Abstract

Western corn rootworm (WCR, *Diabrotica virgifera virgifera* LeConte) is a major corn pest in the United States, causing annual losses of over $1 billion. One approach to protect against crop loss by this insect is the use of transgenic corn hybrids expressing one or more crystal (Cry) proteins derived from *Bacillus thuringiensis*. Cry34Ab1 and Cry35Ab1 together comprise a binary insecticidal toxin with specific activity against WCR. These proteins have been developed as insect resistance traits in commercialized corn hybrids resistant to WCR feeding damage. Cry34/35Ab1 is a pore forming toxin, but the specific effects of Cry34/35Ab1 on WCR cells and tissues have not been well characterized microscopically, and the overall histopathology is poorly understood. Using high-resolution resin-based histopathology methods, the effects of Cry34/35Ab1 as well as Cry3Aa1, Cry6Aa1, and the *Photorhabdus* toxin complex protein TcdA have been directly visualized and documented. Clear symptoms of intoxication were observed for all insecticidal proteins tested, including swelling and sloughing of enterocytes, constriction of midgut circular muscles, stem cell activation, and obstruction of the midgut lumen. These data demonstrate the effects of these insecticidal proteins on WCR midgut cells, and the collective response of the midgut to intoxication. Taken together, these results advance our understanding of the insect cell biology and pathology of these insecticidal proteins, which should further the field of insect resistance traits and corn rootworm management.

## 1. Introduction

Western corn rootworm (WCR, *Diabrotica virgifera virgifera* LeConte) is a major pest of maize (*Zea mays*) in the United States, causing annual losses of over $1 billion [1,2]. WCR eggs hatch in the soil during late spring, and neonate larvae immediately begin feeding on the roots of developing corn plants. Larval feeding damage to roots impairs water and nutrient uptake, results in corn lodging and reduced harvestability, and ultimately reduces overall crop yield. A highly effective means of controlling WCR crop damage in North America is planting transgenic corn hybrids expressing one or more crystal (Cry) proteins from *Bacillus thuringiensis* (Bt) [3]. The Bt crystal proteins currently available in commercialized Bt corn hybrids include Cry3Bb [4], mCry3Aa [5], eCry3.1Ab, and Cry34/35Ab1 [6,7]. Currently, Cry34/35Ab1 are the only commercialized insecticidal proteins not yet impacted by field-evolved WCR populations resistant to Cry3Bb1 maize. WCR populations resistant to Cry3Bb corn are cross resistant to mCry3Aa corn and eCry3.1Ab corn but not to Cry34/35Ab1 corn [8].

The molecular mode of action of Bt toxins has been studied extensively in various insects, and the increasing body of data continues to provide new insights [9,10,11,12]. The current model of Cry protein function begins with activation of Bt proteins by midgut proteases [11,13]. Activated toxins interact with receptors in the midgut epithelial cell membrane and insert to create pores that result in the swelling of epithelial cells due to osmotic stress, and ultimately cell lysis. The loss of these epithelial cells eventually kills the insect through a range of secondary mechanisms. An additional step involving toxin oligomerization prior to insertion has also been proposed [14].

It is possible that every insecticidal protein impacts midgut epithelial cells in a unique way, as there are many potential routes to cause midgut epithelial cell death. In order to determine if ingestion of insecticidal proteins from different classes results in similar or different histopathological symptoms, we decided to evaluate a broad range of insecticidal proteins. Cry3Aa1 is a member of the classical three-domain Bt crystal protein family [15]. Cry34/35Ab1 [6,16,17], Cry6Aa1 [18], and TcdA [19] are all structurally distinct from each other and from three-domain Cry proteins, and have been reported to have different molecular mechanisms of action on WCR [11]. Cry3Aa1, Cry34/35Ab1, and Cry6Aa are all crystal proteins from *Bacillus thuringiensis*; however, TcdA is from *Photorhabdus* spp., and, therefore, represents a very different class of insecticidal proteins [19,20,21].

While some molecular aspects of the mode of action of these proteins are known, at present, relatively little is known about the histopathology of rootworm larvae following ingestion of insecticidal proteins. In general, the effects of Bt toxins on the insect midgut include disruption and loss of microvilli, swelling and vacuolization of midgut enterocytes, and blebbing and cell lysis into the gut lumen. Normally, histopathological studies are carried out on wax sections [22,23]. The typical thickness of wax sections are 5–10 microns [24,25,26]. At this thickness, the fine structure of the small cells of the alimentary system cannot be clearly discerned. On the other end of the spectrum, ultrastructural studies by electron microscopy must be done on ultrathin resin sections [27,28,29]. Here, a single cell, or even part of a single cell, comprises the entire field of view of an electron micrograph (EM). Thus, both wax and EM-level studies fail to provide a detailed picture of cell damage in the broader context of the entire tissue. For the current study, we decided to use EM embedding methods and resins, but at the light level [30]. This type of specimen preparation allows for the study of the fine details of the insect alimentary system, while also providing the overall tissue context. In this report, we describe the effects of four insecticidal proteins on WCR larval internal tissues and cells using high-resolution resin-based histopathology methods. These results allow further understanding of the impact of these proteins on the midgut epithelium, which will drive new discoveries in insect resistance trait discovery and management.

## 2. Results

### 2.1. WCR Anatomy

**Figure 1:**
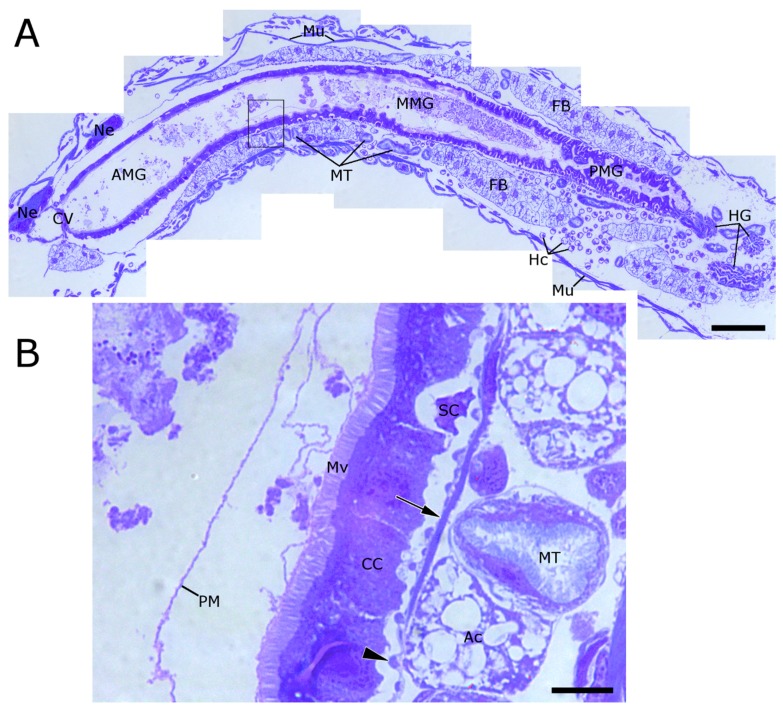
Longitudinal section of western corn rootworm (WCR) showing major anatomical features. (**A**) WCR larvae have a simple “tube within a tube” morphology. The AMG (arrowheads) is composed of a single smooth layer of columnar cells bearing an apical brush border of microvilli. The MMG and PMG regions are characterized by folds and ridges of the gut epithelium. Between the foregut and the anterior midgut is a valve-like structure called the cardiac valve. Fat bodies (FB) and Malpighian tubules (MT) surround the alimentary canal; (**B**) Single layers of peritrophic matrix material are visible in the anterior midgut, but become multi-lamellar in the median and posterior midgut regions. Towards the basal region of the columnar cell layer are pockets of stem cells (SC). The adipocytes (Ac) of WCR larvae contain a large number of oil bodies, which appear very translucent by this preparation method. Malpighian tubules, here seen in cross section, can be seen to have a thick layer of microvilli which appear similar but distinct from those lining the alimentary canal. Surrounding the columnar cells are two layers of muscle fibers, both circular (arrowheads) and longitudinal (arrows). (AMG = anterior midgut; MMG = median midgut; PMG = posterior midgut; CV = cardiac valve; HG = hindgut; Ne = neural tissue; Mu = muscle fibers; Mv = microvilli; CC = columnar cell; PM = peritrophic matrix; SC = stem cells; MT = Malpighian tubules; FB = fat body; Ac = adipocyte; scale bar A = 100 µM, B = 10 µm).

WCR larvae have a worm-like “tube within a tube” morphology (Figure 1A). The alimentary system of WCR is composed of three distinct regions: the foregut, the midgut, and the hindgut. The foregut in this insect appears to be a fairly simple tissue, which functions primarily to pass food from the mouth to the midgut. Between the foregut and the midgut is a valve-like structure called the cardiac valve. The midgut of WCR is composed of a simple columnar epithelium, with microvilli covering the apical region and a membranous labyrinth in the basal portion of the cell. The midgut can be divided into three general sections: the anterior, the median, and the posterior regions. The anterior midgut (AMG) region is immediately adjacent to the foregut, beginning just after the cardiac valve, and the cells of this region are generally very uniform in size and height such that the apical brush border forms a relatively smooth layer lining the lumen (Figure 1A). The lumen of the anterior midgut is relatively dilated and very little peritrophic matrix material is usually visible. In the median midgut (MMG) region, the epithelial cells are flat and thin, and several layers of peritrophic matrix material can be seen lining the lumen (Figure 1B). The posterior midgut (PMG) region is characterized by a distinct increase in folds and ridges of the epithelium; and the lumen tapers to a much smaller diameter in the most distal region (Figure 1A). In the transition from the PMG to the anterior hindgut, the lumen is very narrow and forms a three-dimensional knot-like structure. The hindgut has a highly lobed shape in cross section, and is lined by a chitinous intima.

Outside the alimentary system, the Malpighian tubules can be recognized as smaller, tubular structures lined with fine microvilli on the lumenal surface. The fat body is composed of adipocyte cells containing a large number of lipid droplets, which give these cells a lacy appearance (Figure 1A,B). Skeletal muscle fibers are visible as striated bands running roughly parallel to the axis of the insect, and connect to the segments of the cuticle to provide locomotive force. The nerve cord runs down the length of the larva, on the ventral side of the alimentary system. Various types of hemocytes can be seen in the body cavity.

### 2.2. Cry34/35Ab1

**Figure 2:**
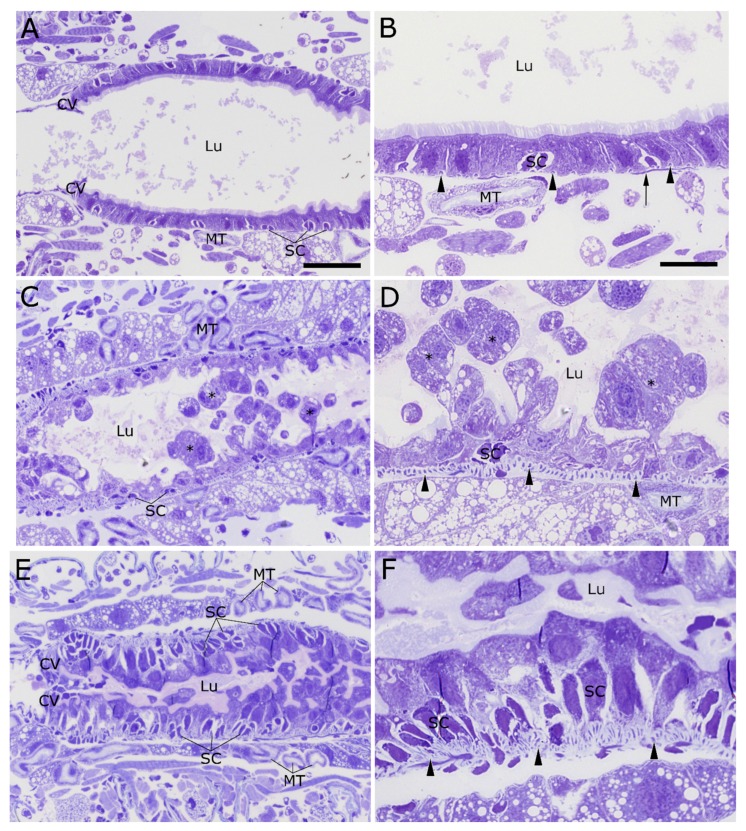
Anterior midgut region of 2nd instar WCR larvae fed Cry34/35Ab1 proteins or buffer. (**A**,**B**) Untreated larvae display a normal midgut epithelial layer, with a smooth layer of columnar cells and an open lumen. Circular muscles fibers (arrowheads) are evenly spaced around the midgut, just exterior to the basement membrane. Longitudinal muscle fibers (arrow) lie just external to the circular muscle fibers. (**C**,**D**) After feeding on diet treated with Cry34/Cry35Ab1 for 24 h, the columnar cells of the AMG show extensive blebbing and sloughing toward the lumen. The apical brush border is no longer continuous, remaining regions are disrupted and reduced, and cell debris is visible in the lumen (asterisks). The stem cell pockets appear relatively unchanged compared to untreated specimens. The bands of circular muscles (arrowheads) surrounding the alimentary canal appear closer together, possibly indicating that they have contracted. (**E**,**F**) At 48 h post-feeding, the lumen of the AMG is nearly completely occluded with cell remnants and swollen columnar cells. The circular muscles (arrowheads) appear to be piling up on each other, possibly as a result of continued intense contractions. The midgut stem cell pockets are also much larger in size, and the cardiac valve appears to have thickened. (CV = cardiac valve; Lu = gut lumen; SC = stem cells; MT = Malpighian tubules; arrowheads = circular muscles; scale bar (**A**,**C**,**E**) = 50 µm; (**B**,**D**,**F**) = 20 µm).

Twenty-four hours after feeding on a diet overlaid with the Cry34/35Ab1 protein pair, the epithelial cells of the anterior midgut appear dramatically different from the untreated control (Figure 2). The cells are clearly disrupted, and cell debris is visible in the midgut lumen (Figure 2C,D). The apical brush border is no longer continuous, and the remaining microvilli are disrupted and reduced (Figure 2C–F). In the untreated insect, a large number of small, widely-spaced circular muscle fibers can be seen surrounding the outer surface of the midgut (Figure 2B, arrowheads). More rarely, longitudinal muscle fibers are observed just exterior to the circular muscle fiber layer. After 24 h of Cry34/35Ab1 feeding, the circular muscle bands have become more prominent and much closer together (Figure 2D, arrowheads). The Malpighian tubules appear relatively normal and unaffected at this time point.

### 2.3. Cry34Ab1 or Cry35Ab1 Alone

**Figure 3:**
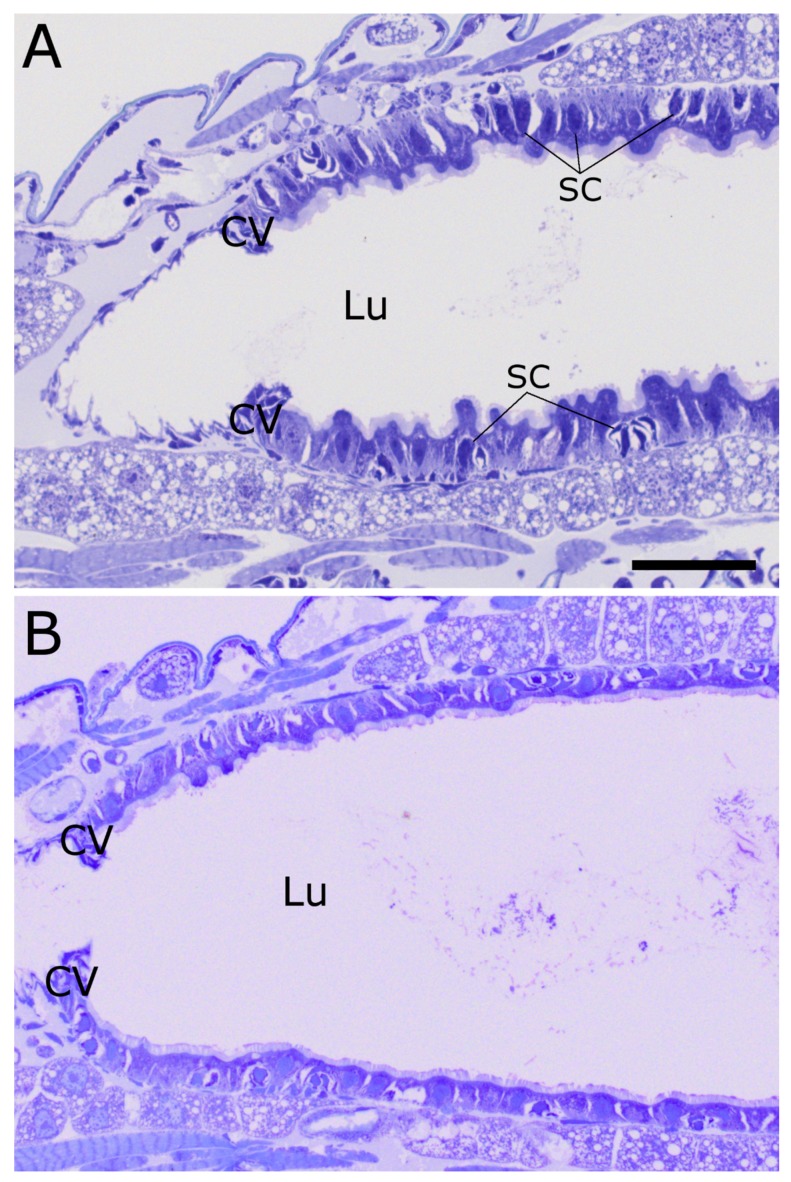
Anterior midgut of WCR larvae fed either Cry34Ab1 or Cry35Ab1 alone at 48 h. (**A**) Ingestion of Cry34Ab1 alone appears to cause some swelling of the apical portion of the columnar cells into the lumen, but very little actual bursting of the cells. The microvilli are still relatively intact and contiguous, but without the obvious cell bursting and/or microvilli shedding seen with the toxin pair, leading to a relatively open lumen. The stem cell pockets appear somewhat enlarged, although not to the extent seen with the toxin pair. The cardiac valve does appear slightly thickened, but the circular muscle bands do not appear heavily contracted; (**B**) The ingestion of Cry35Ab1 alone caused very little disturbance to the morphology of the midgut cells, and had very little impact on the alimentary canal as a whole. (SC = stem cells; CV = cardiac valve; Lu = gut lumen; scale bar = 50 µm).

Ingestion of Cry34Ab1 alone appears to cause swelling of individual midgut epithelial cells, but actual bursting of the cells was not observed (Figure 3A). This swelling may be reducing the continuity of the microvilli on a subset of cells, but the brush border still appears to be relatively intact. The lack of extensive cell bursting and/or microvilli shedding leaves the lumen relatively clear and open, unlike what was seen with Cry34/35Ab1 together. The basal regions of the epithelial cells are more pronounced, possibly as a result of an extension of the basal labyrinth membranes. The individual stem cells of the regenerative clusters appear to have enlarged.

The ingestion of Cry35Ab1 alone appears to have had very little impact on the morphology of the cells of the midgut and the alimentary canal as a whole (Figure 3B). The cells of the anterior midgut appear intact and normal, the microvilli look very similar to the untreated, the circular muscles are relaxed, and the stem cells are small and undifferentiated.

### 2.4. Stem Cell Activation upon Intoxication with Cry34/35Ab1

**Figure 4:**
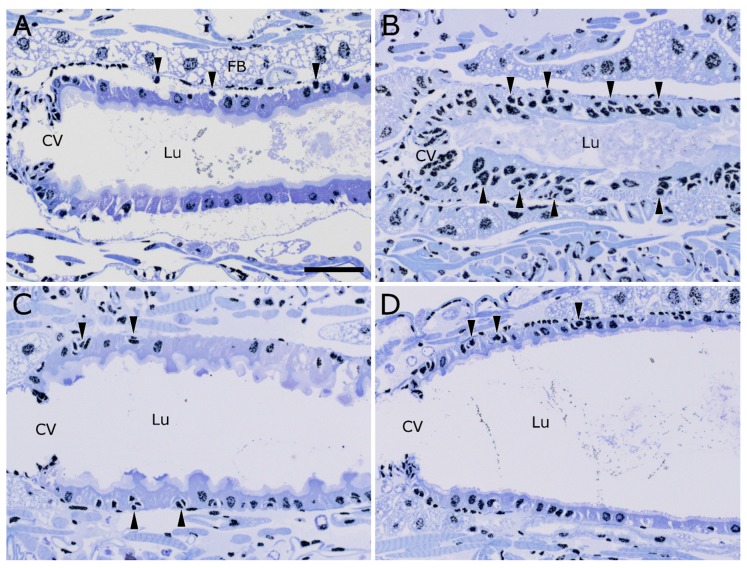
Activation and differentiation of midgut epithelial stem cells following intoxication with Cry34/35Ab1 proteins. Anti-histone H3 immunolabeling (black) on toluidine blue-stained longisections of WCR larvae. (**A**) An untreated WCR larva, showing the normal distribution of cells/nuclei in the midgut epithelium. The midgut epithelium normally consists of a single layer of cells, except for small pockets of small stem cells located on the basal side of the columnar cell layer (arrowheads); (**B**) After feeding on Cry34/35Ab1 for 48 h, multiple layers of cells/nuclei can be seen along the anterior midgut (e.g., arrowheads); (**C**) Larva fed Cry34Ab1 only, showing a single layer of epithelial cells with slightly ruffled apical surfaces, but with apparently non-activated stem cell pockets (arrowheads); (**D**) Larvae fed Cry35Ab1 only appear very similar to the untreated, showing very little perturbation of the epithelial cells and no obvious activation of the stem cell pockets (arrowheads). (CV = cardiac valve; Lu = gut lumen; scale bar = 50 µm).

In order to more clearly identify activated stem cells/developing epithelial cells in the sections, nuclei were labeled with an anti-histone H3 antibody (black) in addition to the toluidine blue staining (Figure 4). In untreated larvae, the epithelial cells lining the AMG form a single layer, as demonstrated by the placement of nuclei (Figure 4A). There are some occasional smaller, more compact cells toward the basal surface of the epithelial cells that are likely midgut epithelial stem cells (Figure 4A, arrowheads). In WCR larvae that have been treated with Cry34/35Ab1 for 48 h, multiple layers of nuclei can be seen in the midgut epithelium (Figure 4B, arrowheads). The nuclei closest to the lumen are likely from damaged and dying cells, while the nuclei closer to the basement membrane are stem cells and newly-developing replacement epithelial cells. In larvae fed with only Cry34Ab1 (Figure 4C) or Cry35Ab1 (Figure 4D), the midgut epithelial cells remain as a single layer, with no obvious stimulation of stem cell multiplication and differentiation, similar to that of the untreated.

### 2.5. Other Insecticidal Proteins

**Figure 5:**
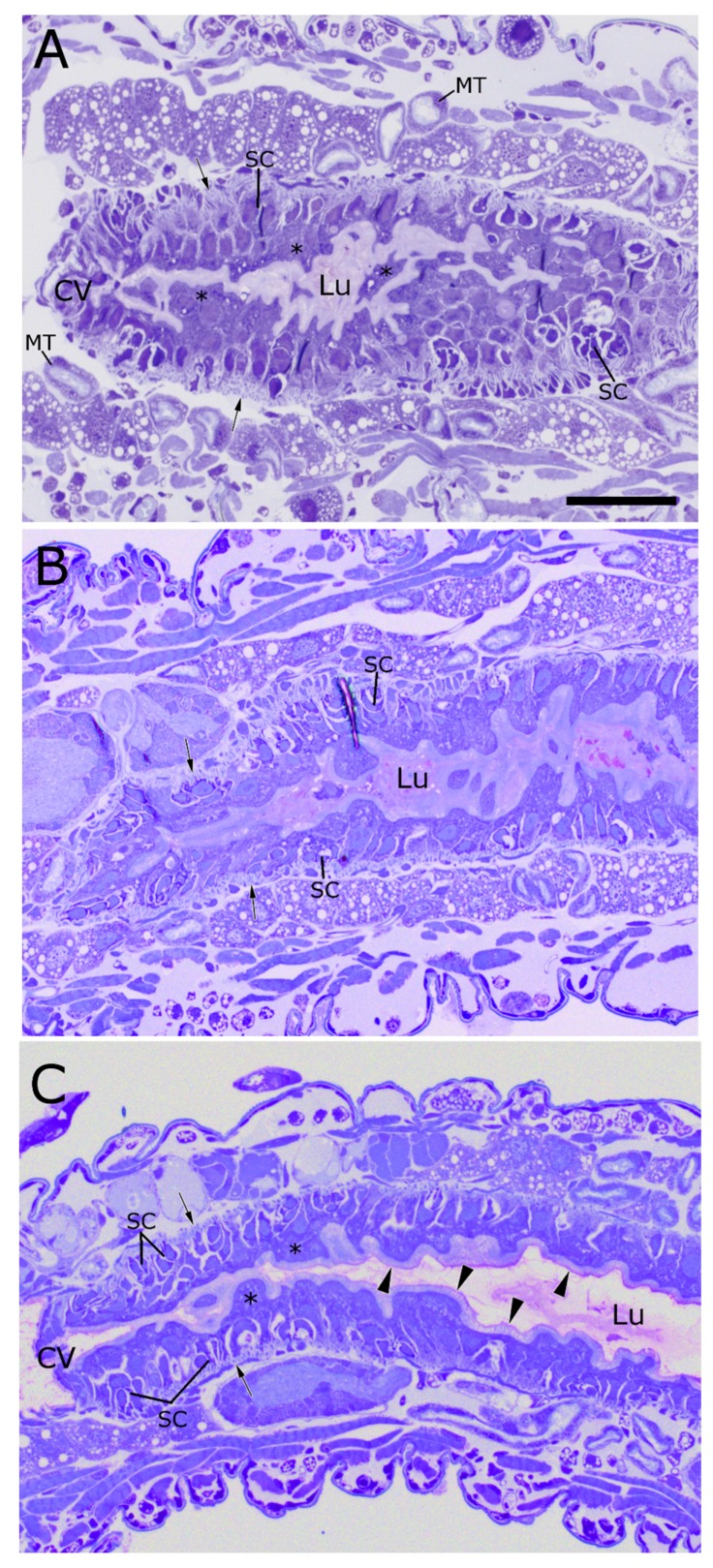
AMG of WCR larvae fed with various insecticidal protein toxins, after 48 h of continuous exposure. (**A**) Ingestion of Cry3Aa induces damage similar to that induced by Cry34/35Ab1, primarily, a massive lysis and shedding of AMG columnar cells (asterisks), stem cell activation and growth, contraction of circular muscle bands (arrows), and occlusion of the midgut lumen by cell debris; (**B**) Ingestion of Cry 6Aa results in damage similar to that induced by Cry34/35Ab1, including overall midgut cell disorder, columnar cell blebbing and lysis, contraction of the circular muscle bands (arrows), and stem cell activation. The lumen is completely full of an apparently non-cellular material, possibly columnar cell lysates and/or secretions. The stem cells appear to be activated and have begun differentiating into replacement enterocytes; (**C**) TcdA: the stem cells in the AMG appear to have been stimulated and are in advanced stages of columnar cell replacement. Remnants of the columnar cells can still be seen lining the gut lumen (asterisks). The apical microvilli of the columnar cell remnants appear to be relatively intact (arrowheads). The circular muscles appear contracted (arrows), but the lumen does not appear to be entirely occluded. (CV = cardiac valve; SC = stem cells; Lu = gut lumen; MT = Malpighian tubules; scale bar = 50 µm).

*Cry3Aa1*. Treatment of WCR larvae with Cry3Aa results in an occluded lumen that appears very similar to what was observed for Cry34/35Ab1 (Figure 5A). The columnar cells of the AMG have lysed, and their contents have been released into the lumen (Figure 5A, asterisks). The midgut stem cells have been activated and are beginning to differentiate into new columnar cells to replace the damaged and dying columnar cells. The circular muscle bands are contracted (Figure 5A, arrows). This lysis and shedding of midgut cells, the replacement of these damaged columnar cells by stem cell activation and growth, and the contraction of the circular muscle bands has caused a near-total occlusion of the midgut, just as was seen with Cry34/35Ab1 intoxication. The Malpighian tubules appear normal.

*Cry6Aa1*. The lumen of the AMG appears to be filled with material composed of shed microvilli and other cellular contents (Figure 5B), similar to what was seen with Cry34/35Ab1 and Cry3Aa. The epithelial cells of the midgut appear to be swollen and in some cases have ruptured. There is activation and growth of midgut stem cells, and the circular muscle bands are contracted (Figure 5B, arrow). Cry6Aa1 appears to lead to the near-total closure of the anterior midgut lumen, as seen for Cry34/35Ab1 and Cry3Aa.

*TcdA.* TcdA intoxication leads to a subtly different pattern of damage than what was observed with the Cry toxins in this study. Unlike the Cry proteins, TcdA intoxication appears to damage the midgut epithelium without destroying the apical microvilli. The columnar cells appear as a ruffled layer covering a nearly uniform layer of developing midgut stem cells (Figure 5C). However, even at these late stages of dying columnar cell replacement, the apical microvilli of these severely damaged cells appear to be relatively intact (Figure 5C, arrowheads). The circular muscle bands appear to be contracted (Figure 5C, arrows), as they do after treatment with Cry34/35Ab1; however, the lumen of the midgut does not appear to be entirely occluded.

## 3. Discussion

Bt proteins are a valuable defense mechanism for maize against WCR. Once consumed by WCR, Cry proteins appear to disrupt the anterior midgut epithelial cells, which induces circular muscle contraction and stem cell activation. These events progress to the point of nearly complete occlusion of the midgut lumen and, ultimately, the death of the insect. Many previous reports on the exposure of Cry proteins to insect midgut cells have focused on the few hours immediately following ingestion of Cry proteins [27,31,32]. The main phenomena at these early time points are usually observed at the TEM level, and are described as blebbing of microvilli, swelling of mitochondria, and dilation of intracellular spaces, culminating in cell lysis. Also, previous histopathological studies have tended to focus on lepidopteran species. Here, we have decided to focus on the later stages (48 h PI) of the Cry protein intoxication process and to compare the action of several insecticidal proteins on the midgut epithelium in WCR.

From a technical standpoint, cross sections of insect alimentary systems are relatively straightforward to prepare. Longisections, however, result in a better understanding of midgut cell variation along the length of the gut than do cross sections. The morphology of the midgut cells change along the length of the gut, such that the appearance of midgut cells in a given cross section are highly dependent on the exact location along the midgut from which that cross section was made. In addition to this, longisections allow for a better visualization of the circular muscle fibers, the contraction of which were found to be one of the main hallmarks of the insecticidal protein intoxication process in WCR.

The integrity and correct functioning of the alimentary system is critical for the growth and survival of insect larvae. The ingestion of the Cry34/35Ab1 protein pair causes significant damage to the cells of the anterior midgut, including swelling and lysis of the midgut epithelial cells, shedding of microvilli and other cell debris, and the constriction of the circular muscle fibers surrounding the alimentary canal. After 48 h post-feeding, the large amount of cell swelling, debris, damage to the cardiac valve, and constriction of the circular muscle fibers appears to totally occlude the lumen of the midgut. The observed occlusion of the midgut lumen would render further ingestion of food difficult or impossible, leading to the death of these insects by starvation and/or dehydration. We have found no evidence that Cry protein intoxication of WCR leads to death by sepsis, though these studies were all carried out on lab-reared insects, which may not be totally reflective of conditions in the field. Interestingly, the effects of Cry3Aa and Cry6A, which are also pore forming proteins, on WCR were very similar to those seen with Cry34/35Ab1. This evidence indicates that the death of midgut enterocytes by plasma membrane pore-forming proteins leads to similar midgut tissue damage, irrespective of the different midgut binding sites utilized by these disparate proteins.

Cry34Ab1 is a protein of approximately 14 kDa with features of the aegerolysin family (Pfam06355) of proteins that have known membrane disrupting activity, while Cry35Ab1 is an approximately 44 kDa member of the toxin_10 family (Pfam05431) that includes other insecticidal proteins such as the BinA/BinB binary toxin [16]. Recent studies [33] on WCR midgut membrane receptor identification and interaction with Cry34/35Ab1 indicate that Cry34Ab1 significantly enhances the binding of Cry35Ab1 to brush border membrane vesicles (BBMV) of WCR larvae. However, when applied separately, Cry34Ab1 and Cry35Ab1 did not cause obvious damage to the midgut epithelium or circular muscle fiber contractions. Cry34Ab1 did cause some ruffling of the midgut epithelium and stimulation of stem cell differentiation, but Cry35Ab1 did not. These results are consistent with reports of the action of these individual proteins on southern corn rootworm in traditional bioassays [34]. Although subtle, this observation may be further evidence of both an interaction between Cry34Ab1 with a receptor on the midgut brush border membrane, and the lack of direct binding of Cry35Ab1 to the brush border membrane.

In the normal course of the development of rootworm larvae, old or damaged columnar cells are replaced by the development of midgut stem cells into new columnar cells [35]. The ingestion of Cry34/35Ab1 leads to extensive damage to the cells of the midgut epithelium (Figure 2). This damage can be repaired by the activation and differentiation of midgut stem cells to become new enterocytes (Figure 4). It has been suggested that one mechanism of resistance to Cry protein intoxication is an increased healing response [36,37].The direct visualization of nuclei in situ in midgut sections may be a valuable method to evaluate resistant colonies for the existence of rapid healing response-based resistance mechanisms.

TcdA appears to cause swelling of the anterior midgut epithelial cells and activation of stem cell multiplication and differentiation, but relatively less shedding of microvilli than the Cry toxins. TcdA proteins from *Photorhabdus* are endocytosed by midgut cells and then form channels in the endosome membrane [19,38,39]. As described in the literature, the TcdA component of the toxin complex functions to inject the TcdB and TcdC subunits into the cell cytoplasm, where these subunits interfere with actin filaments and ultimately cause the death of the cell. Although cell death is usually accomplished through the combined action of three different members of the complete toxin complex, it has been shown that the TcdA protein is toxic by itself [21,40]. In WCR, intoxication by TcdA caused the circular muscles to contract around the AMG and the midgut stem cells to be activated, both of which were also seen in the Cry-treated examples. However, the brush border of the midgut epithelium appeared more intact, and the lumen of the TcdA-intoxicated larvae appeared to be less obstructed than what was observed for the other Cry toxins. This may be due to the lesser amount of cell lysis and sloughing noted following TcdA intoxication. Further ultrastructural studies comparing the impacts of toxin complex and Cry proteins on WCR midgut cells may yield additional insights.

In summary, we have provided an in-depth histological characterization of the WCR larva. We have also demonstrated the impact of the Cry34/35Ab1 protein pair on the midgut of WCR. These impacts were shown to be primarily swelling and lysis of midgut epithelial cells in the midgut lumen, stimulation of regenerative stem cells, and contraction of the circular muscle fibers. These features were shown to be the same as other Cry-proteins, and similar to the damage induced by the TcdA toxin. Taken together, these results represent a foundation that future studies of the action of insecticidal proteins can be built upon.

## 4. Materials and Methods

*Protein preparation.* Cry34/35Ab1. Cry protein inclusion bodies produced from recombinant *Pseudomonas fluorescens* clones MR1253 and MR1636 (expressing Cry34Ab1 and Cry35Ab1 proteins, respectively) were resuspended separately in 25 mL of 100 mM sodium citrate buffer, pH 3.0, in a 50-mL conical tube [17]. The tubes were placed on a gently rocking platform (Vari-Mix™, Thermo Fisher Scientific, Waltham, MS, USA) at 4 °C overnight to extract full-length Cry34Ab1 and Cry35Ab1 proteins. The extracts were centrifuged at 30,000× *g* for 30 min at 4 °C and supernatants containing full-length Cry proteins were retained. The supernatant of Cry34Ab1 was then concentrated using a centrifugal concentrating device with a 10 kDa MWCO (molecular weight cut off). The concentrated sample was buffer exchanged via PD-10 into 20 mM sodium citrate pH 3.5. After the concentration of Cry34Ab1 was determined by gel densitometry, it was stored at 4 °C for WCR larval feeding. For Cry35Ab1, the supernatant was then purified over a 5 mL HiTrap™ SP cation exchange column (GE Healthcare Bio-sciences Corp., Piscataway, NJ, USA). Fractions containing Cry35Ab1 were pooled and concentrated with a 10 kDa MWCO Amicon concentrator. The sample was then dialyzed overnight against 20 mM sodium citrate pH 3.5. Sample concentration was determined by gel densitometry with bovine serum albumen (BSA) as a standard and were ready for insect feeding.

Cry3Aa and Cry6Aa. These proteins were prepared as previously described [31]. Briefly, the protein inclusions from recombinant *P. fluorescens* strains MR832 and DPf13032, expressing Cry3Aa and Cry6Aa, respectively, were resuspended in 100 mM sodium carbonate buffer, pH 11.0 for Cry3Aa, or in 50 mM CAPS [3-(cyclohexamino) 1-propanesulfonic acid] buffer, pH 10.5 for Cry6Aa. Full-length Cry protoxins were extracted in the basic buffer as described above. The supernatant was then concentrated using a centrifugal concentrating device with a 30 kDa MWCO. The concentrated sample supernatants were purified using a 5 mL HiTrap™ Q HP ion exchange column (GE Healthcare Bio-sciences Corp., Piscataway, NJ, USA) and subjected to buffer exchange via a PD-10 column (GE Healthcare Bio-sciences Corp., Piscataway, NJ, USA) into 10 mM CAPS pH 10. After the protein concentrations were determined by gel densitometry, they were ready for insect feeding assays.

TcdA. Cell paste from *Pseudomonas fluorescens* (Pf) was resuspended in 5× the volume of extraction buffer (50 mM sodium carbonate pH 9.2 + 1 mg/mL lysozyme + 700 μL His-tagged protease inhibitor cocktail (Sigma-Aldrich, St. Louis, MO, USA)). The suspension was mixed at 4 °C for 20 min followed by sonication (Branson Sonifier 450 with flat tip) three times for 3 min per cycle with 5 min of rest between cycles. The suspension was then clarified via centrifugation at 20,000× *g* for 25 min at 4 °C. The clarified supernatant was loaded onto a 50 mL Q Sepharose Fast Flow column equilibrated in Buffer A (50 mM sodium carbonate pH 9.2). The column was eluted using a linear gradient from 0–100% Buffer B (50 mM sodium carbonate pH 9.2 + 0.5 M NaCl) over 2000 mL at 15 mL/min. The fractions were analyzed by SDS-PAGE for TcdA and pooled accordingly. The TcdA containing fractions was then precipitated at 50% ammonium sulfate and centrifuged at 20,000× *g* for 25 min at 4 °C. The resulting pellet was resuspended in HIC buffer B (hydrophobic interaction chromatography; 50 mM sodium phosphate pH 7.0 + 1 M ammonium sulfate). The resuspended sample was applied to a HiTrap Octyl column that was equilibrated in HIC buffer B. The column was washed with 10 column volumes of 10% HIC Buffer A (50 mM sodium phosphate pH 7.0 + 100 mM NaCl). The column was then eluted at 100% HIC buffer A for 15 CV. The fractions were analyzed by SDS-PAGE, and TcdA containing fractions were pooled and concentrated via spin concentrators with a 100 kDa MWCO. The concentrated sample was then buffer exchanged via PD-10 into 20 mM sodium phosphate pH 8.0. The final protein concentration was determined by gel densitometry.

*Insect bioassay and exposure*. The non-diapause WCR used in this study was obtained from a commercial supplier, Crop Characteristics Inc., Farmington, MN, USA. The non-diapausing WCR colony was started in 1999, approximately, from Minnesota wild diapausing adults, and they have been raised for about 100 generations from the wild type. The materials and methods of WCR egg incubation, egg surface sterilization, and diet overlay bioassay were previously described [34]. In this study, WCR larvae that were approximately 48-h old were used for insect bioassays. Sixteen larvae were exposed at each time point collection per individual protein or designated buffer. About ten larvae were randomly collected per treatment for the histopathology study. Treatments were comprised of exposure to 16.5 µg/cm^2^ full length (FL) Cry34Ab1 and 16.5 µg/cm^2^ truncated (TR) Cry35Ab1 for a total 33 µg/cm^2^ of 1:1 (W/W) ratio mixture of the two proteins. FL Cry34Ab1 and TR Cry35Ab1 treatments were applied separately at 50 µg/cm^2^. In addition, 350 µg/cm^2^ of TR Cry3Aa, 33 µg/cm^2^ of FL Cry6Aa, and 10 µg/cm^2^ of TcdA treatments were included as well. These doses were chosen based upon their different potency on WCR larvae in diet-based bioassay to ensure that they cause gut tissue damages of the insects. Buffer control for Cry34/35Ab1 was 20 mM sodium citrate pH 3.5 for Cry3Aa, for Cry6Aa it was 10 mM CAPS pH 10, and for TcdA it was 20 mM sodium phosphate pH 8.0.

*Specimen collection and preparation for histopathology*. Insects were collected in 24-hour increments. Once collected, insects were fixed in 4% formaldehyde (from sealed ampules; Polysciences, Inc., Warrington, PA, USA) in 10 mM phosphate buffered saline, with 1:10,000 Silwet L-77 (Lehle Seeds, Round Rock, TX, USA), vacuumed until specimens sank, dehydrated in a graded series of ethanol (25%, 50%, 75%, and 100%), and infiltrated in a graded series of LR White resin (Polysciences, Inc., Warrington, PA, USA) [30]. Larvae were transferred into flat bottom polyethylene embedding capsules (Ted Pella, Redding, CA, USA), topped up with fresh LR White resin, and heat polymerized for 3 h at 50 °C. A relatively large number of each specimen type were polymerized into blocks so that WCR larvae in the ideal orientation for longi-sectioning could be selected. The selected WCR larvae were then sectioned (500 nm), stained with Toluidine Blue O, and mounted with Polymount-Xylene (Polysciences, Inc., Warrington, PA, USA).

*Immunolocalization*. For each sample type, 500 nm thick LR White sections were generated on a Leica UC7 ultramicrotome (Leica Microsystems, Buffalo Grove, IL, USA). Sections were placed on SuperFrost Plus slides (Fisher Scientific, Pittsburgh, PA, USA) and allowed to dry on a slide warmer. Slides were processed on a Ventana Discovery Ultra immunostainer (Roche Diagnostics, Indianapolis, IN, USA). The histone antibody (ab1791, Abcam, Cambridge, MA, USA) was diluted 1:200 in Ventana Antibody Diluent 250 (Roche Diagnostics, Indianapolis, IN, USA); the antibody was incubated on the slides for 32 min at 37 °C. The secondary antibodies (anti-Rabbit HQ-HRP Ventana Discovery system, Roche Diagnostics, Indianapolis, IN, USA) were then applied, and each component was incubated for 16 min at 37 °C. Finally, Ventana Discovery Silver (Roche Diagnostics, Indianapolis, IN, USA) was applied and incubated for 12 min at room temperature. Slides were washed in soapy water, dried, dipped briefly in xylene, and mounted with Polymount-Xylene (Polysciences, Inc., Warrington, PA, USA).

*Imaging*. Image data were captured using LAS software (version 4.6) on a Leica DM5000B upright microscope, equipped with a Leica DFC 7000T camera (Leica Microsystems, Buffalo Grove, IL, USA). Figure panels were created with GIMP (v2.8.16). Minor contrast and color balance adjustments were done with GIMP.
